# Synthesis, Characterization, and Evaluation of the Antimicrobial and Anticancer Activities of Zinc Oxide and Aluminum-Doped Zinc Oxide Nanocomposites

**DOI:** 10.3390/ph17091216

**Published:** 2024-09-16

**Authors:** Muhammad Asif, Muhammad Fakhar-e-Alam, Muhammad Tahir, Farah Jamil, Hassan Sardar, Javed Rehman, Kholood A. Dahlous

**Affiliations:** 1 Department of Physics, Government College University Faisalabad, Faisalabad 38000, Pakistan; 2 Department of Chemistry, Quaid-e-Azam University, Islamabad 45320, Pakistan; 3 Department of Applied Chemistry, Government College University Faisalabad, Faisalabad 38000, Pakistan; 4 State Key Laboratory of Metastable Materials Science and Technology, School of Materials Science and Engineering, Yanshan University, Qinhuangdao 066004, China; 5 MEU Research Unit, Middle East University, Amman 11831, Jordan; 6 Department of Chemistry, College of Science, King Saud University, Riyadh 11451, Saudi Arabia

**Keywords:** ZnO NPs, Al-doped ZnO NCs, antimicrobial, anticancer activities

## Abstract

In this research, we developed undoped and aluminum-doped zinc oxide for antimicrobial and anticancer activities. This study focuses on the synthesis, characterization, and biological activities of zinc oxide nanoparticles (ZnO NPs) and aluminum-doped zinc oxide nanocomposites (Zn_1−x_Al_x_O NCs) at varying concentrations (x = 0, 0.25, 0.5, and 0.75 wt%) using the coprecipitation method. Various characterization techniques such as XRD, UV-Vis, FTIR, EDX, and SEM were performed to analyze the crystal structure, optical properties, functional group identification, elemental composition, and surface morphology. The antimicrobial activity test showed that Zn_0.75_Al_0.25_O NCs exhibited the strongest inhibition zone against *Bacillus cereus* compared to *Staphylococcus aureus* > *Pasteurella multocida* > *Escherichia coli*. Moreover, the cytotoxicity and cell viability of liver cancer (HepG-2), breast cancer (MCF-7), ovarian cancer (SKOV3), and normal liver cell lines) were evaluated using the MTT assay, demonstrating that Zn_0.75_Al_0.25_O NCs not only enhance cell destruction but also show low cytotoxicity and high biocompatibility at low concentrations. These results suggest that Zn_0.75_Al_0.25_O NCs could be a promising candidate for in vivo anticancer applications and should be further investigated.

## 1. Introduction

Nanotechnology offers numerous advantages in every field of life, including information technology, medical technology, energy, global security, environmental science, and food safety. However, its impact is particularly effective in the field of nanomedicine, where it holds great potential for cancer diagnosis and treatment. Cancer, a leading cause of mortality and healthcare expenditure, affects millions globally, manifesting in various forms such as breast, liver, lung, and brain cancer [1,2,3].

According to a 2020 WHO report, cancer is the second foremost reason for death globally, resulting in over 10 million deaths annually. Liver cancer (HepG-2) is one of them, and unfortunately, it is on the rise [4,5]. Notably, 85% of cancer cases occur in developing nations. Pakistan has the highest death rate of liver cancer among Asian nations [6,7]. The major risk factors for HepG-2 cancer include infection with human papillomavirus (HPV) and hepatitis (HCV, HBV), as well as exposure to fatty acids, contaminated food, nicotine, and alcohol, which contribute to 25% of liver cancer deaths. However, these factors may vary from country to country [8,9]. Moreover, bacterial infections and their resistance have severely obstructed public health, causing widespread morbidity and mortality [10]. The treatment of bacterial infections often involves traditional medicines, which can have harmful side effects due to overuse and misuse, ultimately impacting patients’ health and well-being [11,12,13].

Metal oxide nanoparticles (MO NPs) and their nanocomposites (especially ZnO NPs, Al_2_O_3_ NPs, and their nanocomposites) have demonstrated high efficiency against antimicrobial resistance (AMR), multidrug resistance (MDR), as well as in cancer diagnostics and treatment, and inhibiting both Gram-positive and Gram-negative bacteria [14,15].

A wide range of MO NPs, including ZnO NPs, manganese dioxide, magnesium oxide, aluminum oxide, silver oxide, and selenium oxide nanoparticles, as well as their nanocomposites, have gained significant attention in the biomedical field for applications such as nanomedicine, drug delivery systems (DDS), biosensing, photothermal therapy (PTT), hyperthermia therapy, photodynamic therapy (PDT), diagnostics, and cancer treatment. This interest is driven due to their exceptional physiochemical and biological properties [16,17,18,19,20,21]. For example, Rana et al. [22] described that ZnO NPs and Al-doped ZnO NCs exhibit broad-spectrum antimicrobial activity and promising efficacy against Gram-negative and Gram-positive bacteria. They also exhibit enhanced cytotoxicity toward various cancerous cell lines, including liver, breast, lung, and colon cancer cells. It can also boost cell death by inhibiting cancer growth cells. Yu et al. [23] reported that Al-doped ZnO NCs show great antibacterial activity against various bacterial strains, including *E. coli* and *P. aeruginosa*, especially for *S. aureus*. Moreover, they can also decrease antimicrobial resistance (AMR). Eddy et al. [24] described that Al-doped ZnO NCs exhibited broad-spectrum antimicrobial activity and promising efficacy against Gram-negative and Gram-positive bacteria. These nanocomposites can enhance cytotoxicity, reduce toxicity, and have minimal side effects on various cancerous cell lines, including liver, breast, lung, and colon cancer cells. Also, it was found to boost cell death while inhibiting cancer cell growth [12].

There are several reputable techniques for developing MO NPs, such as auto-combustion, coprecipitation, sol–gel, or hydrothermal methods, etc. [25,26]. These methods are ideal due to their simplicity, ease of use, eco-friendliness, and ability to fabricate nanoparticles with the desired morphology. Furthermore, the size, shape, and properties of nanoparticles can be easily modified by adjusting reaction parameters, including temperature and time [27,28,29].

In this research, undoped and aluminum-doped zinc oxide were successfully fabricated and characterized using various characterization techniques. These nanocomposites not only inhibit bacterial growth and enhance cell destruction but also show low cytotoxicity and high biocompatibility at low concentrations. Additionally, to address the problems of AMR and MDR barriers using these nanomaterials. These findings suggest that Zn_0.75_Al_0.25_O NCs could be promising candidates for in vivo anticancer applications.

## 2. Results and Discussions

### 2.1. XRD Analysis

**Figure 1:**
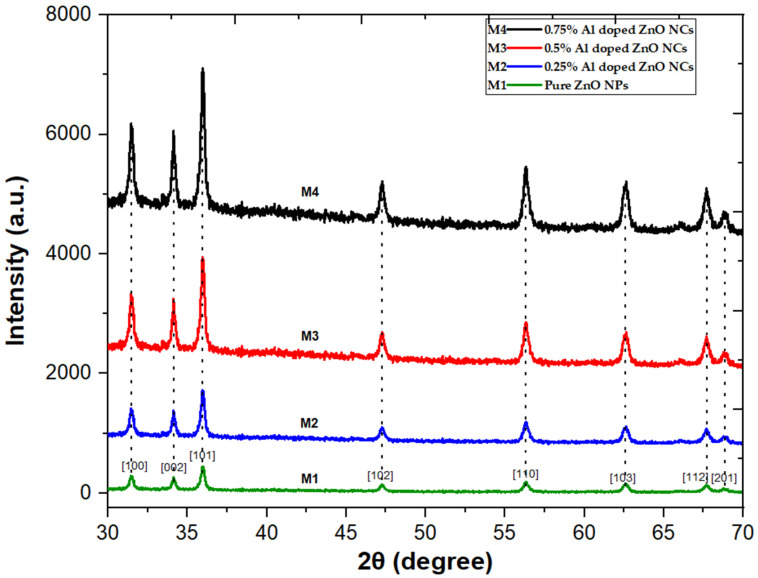
XRD spectrum of ZnO NPs and Al-doped ZnO NCs at varying concentrations 0.25%, 0.5%, and 0.75%.

XRD analysis was employed to investigate the structural properties of ZnO NPs and Al-doped ZnO nanocomposites fabricated at varying concentrations (0.25%, 0.5%, and 0.75%). The XRD pattern revealed no additional phases, and the intensity of the samples increased by increasing aluminum concentration, as shown in Figure 1. The XRD patterns of ZnO NPs and Al-doped ZnO NCs showed three distinct peaks at 31.5°, 34.08°, and 36.01°, corresponding to the Miller indices (100), (002), and (101), respectively. These peaks matched well with the JCPDS card no. 36-1451, verifying the hexagonal wurtzite phase structure of ZnO NPs and Al-doped ZnO NCs with complete formation and no additional peaks. Moreover, low-intensity peaks were observed at 47.33°, 56.39°, 62.69°, 67.75°, and 68.89°, corresponding to the Miller indices (102), (110), (103), (112), and (201), respectively [16,19]. Table 1 shows that the crystallite size of Al-doped ZnO NPs does not change significantly with increasing Al concentration. The non-significant changes were observed in crystallite size with increasing Al concentration, which can be significant in various applications, such as electronic and optoelectronic devices, energy storage devices, and photocatalytic activities, particularly in biomedical applications. Furthermore, due to their small size, nanoparticles can be effectively used for targeted drug delivery [26,27]. The average crystallite sizes of ZnO NPs and Al-doped ZnO NCs were calculated using Scherrer’s Equation (1).

(1) $$C.\;S=\frac{k\lambda }{\beta cos\theta }$$

where,

*C.S* = crystallite size;

*k* = shape factor (usually its value is 0.94);

λ = 0.154 nm, X-ray wavelength;

β = FWHM (full-width half maximum) in radian;

Cosθ = Bragg angle in degrees.

**Table 1:** ZnO NPs and Al-doped ZnO NCs at varying concentrations (0.25%, 0.5%, and 0.75%).

(h, k, l) Miller Indices	2θ (Angle of Diffraction)	*C.S* (nm)
ZnO NPs		
100	31.5°	39.37
101	36.01°	45.25
110	56.39°	47.1
112	67.75°	44.54
Zn_0.75_Al_0.25_O NCs		
100	31.5°	36.67
101	36.01°	41.65
110	56.39°	43.71
112	67.75°	42.64
Zn_0.5_Al_0.5_O NCs		
100	31.5°	35.17
101	36.01°	39.29
110	56.39°	42.31
112	67.75°	40.54
Zn_0.25_Al_0.75_O NCs		
100	31.5°	32.87
101	36.01°	33.26
110	56.39°	35.58
112	67.75°	31.29

### 2.2. Fourier Transformation Infrared Spectroscopy

**Figure 2:**
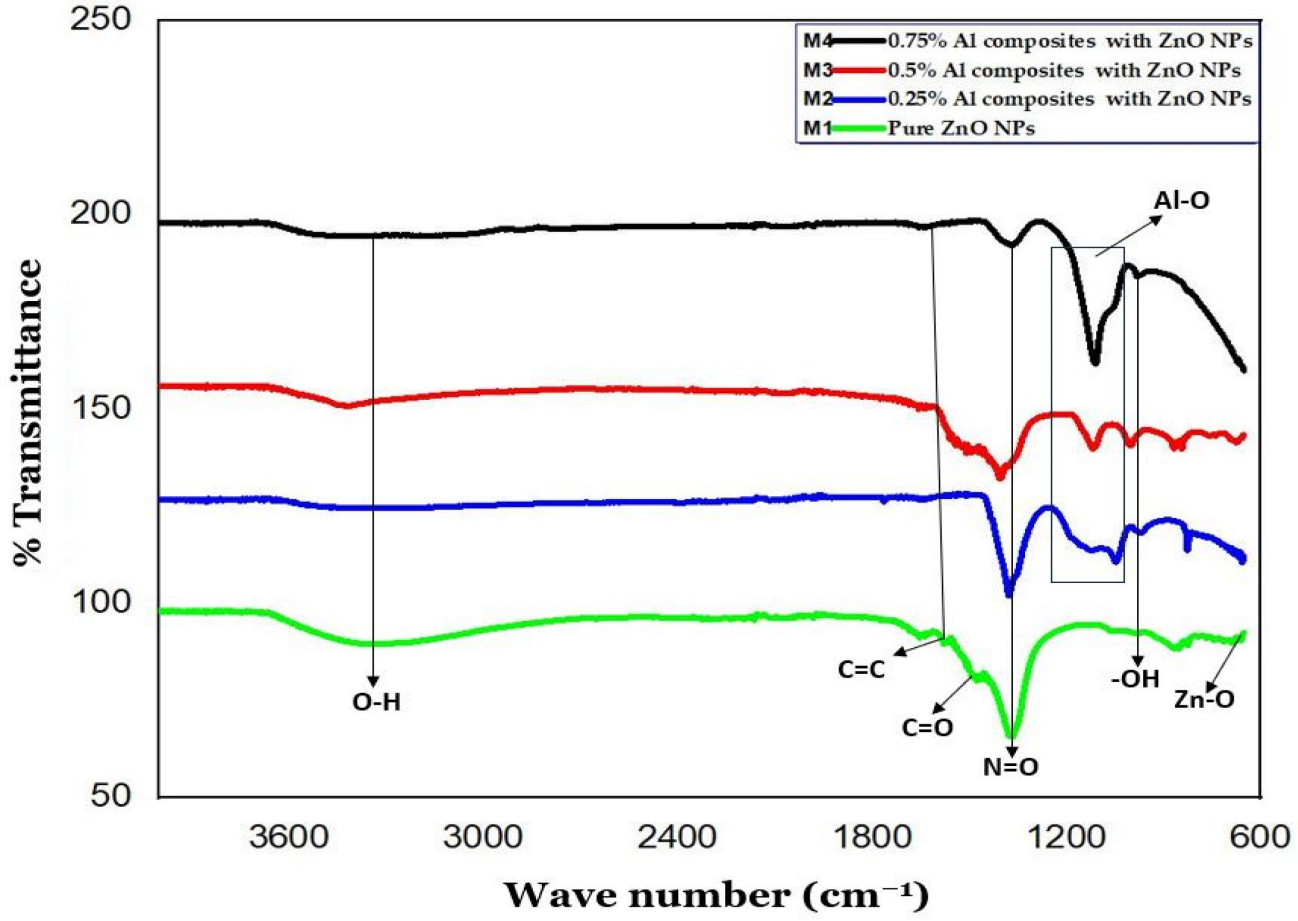
FTIR spectrum of ZnO NPs and Al-doped ZnO NCs at varying concentrations 0.25%, 0.5%, and 0.75%.

Figure 2 displays the FTIR spectra of ZnO NPs and Al-doped ZnO NCs at varying concentrations (0.25%, 0.5%, and 0.75%) over the 400–4000 cm^−1^ range. The FTIR spectra disclose the Zn-O stretching mode, thereby representing a band between 441 and 665 cm^−1^, confirming the formation of ZnO NPs. Moreover, the broad band at 3349 cm^−1^ corresponds to the O-H stretching mode of the hydroxyl group, while peaks at 890 cm^−1^ and 3349 cm^−1^ indicate -OH bending and stretching vibrational modes. These peaks originate from the H_2_O during Zn-O formation and moisture contents in the atmosphere. However, the intensity and magnitude of the –OH emission at 3349 cm^−1^ vary due to the use of different reducing agents. The peaks at 1422 cm^−1^ and 1642 cm^−1^ correspond to C=O and C–H vibrational modes, respectively, which are due to the presence of aldehyde or ketone groups partially substituted by zinc source nitrate ions (Zn(NO_3_)_2_). The absorption at 1376 cm^−1^ suggests the presence of an aromatic ring at a moderate level. The observed peaks at 1642, 1460, 1422, 1376, and 865 cm^−1^ are associated with the asymmetrical and symmetrical zinc carboxylate [29,30,31].

### 2.3. UV-Visible Spectroscopy

**Figure 3:**
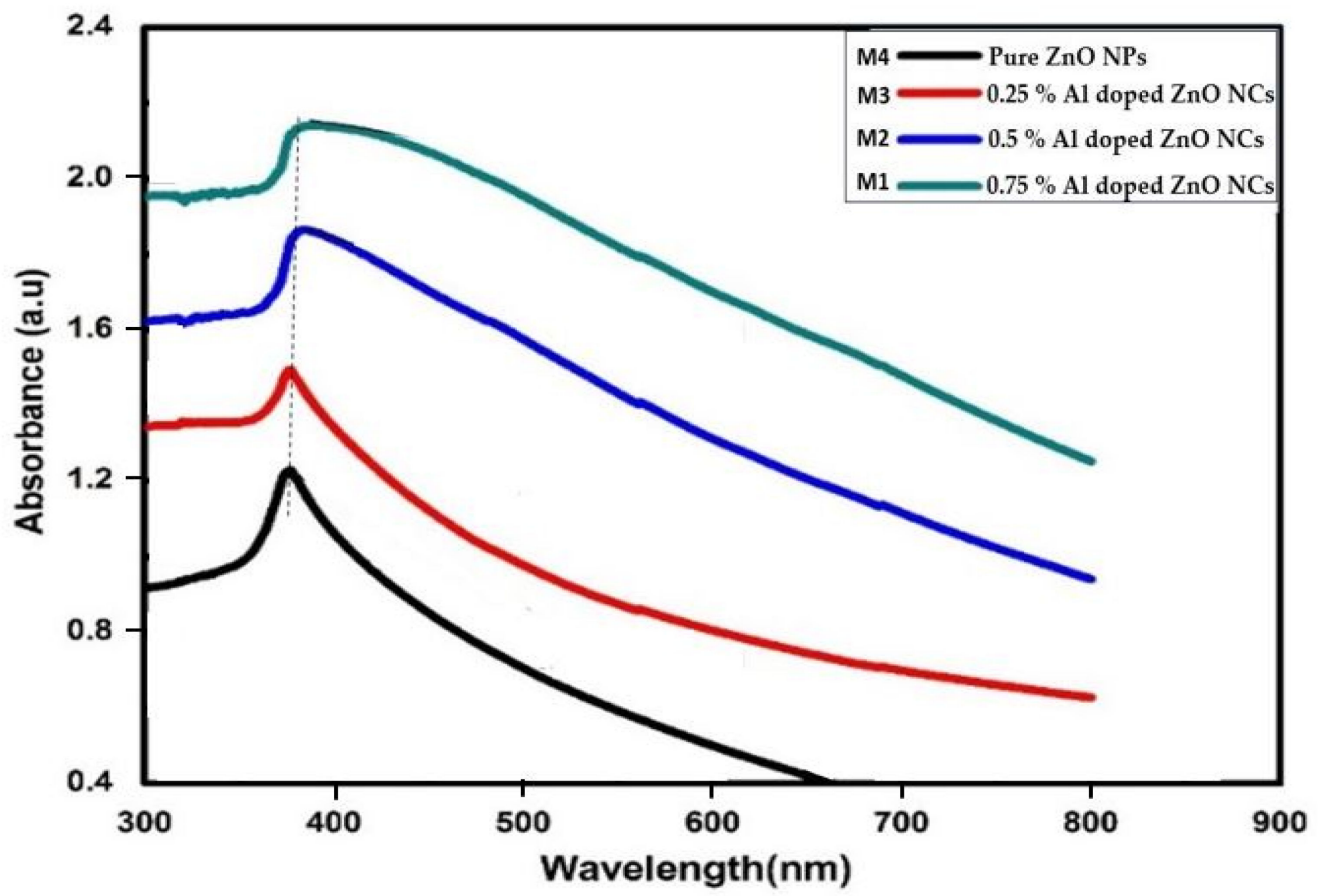
UV-visible spectrums of ZnO NPs and Al-doped ZnO NCs at varying concentrations 0.25%, 0.5%, and 0.75%.

The UV-visible spectra in Figure 3 illustrate the absorption bands of ZnO NPs and Al-doped ZnO NCs at varying concentrations (0.25%, 0.5%, and 0.75%). The presence of aluminum causes a longer wavelength (red shift). Because of their high exciton binding energy (60 mV), ZnO NPs and their Al-doped ZnO NCs exhibit substantial exciton absorption in their spectra, even when synthesized at ambient temperature. Specifically, the absorption peak of ZnO NPs and Al-doped ZnO NCs occurs at 363 nm and ≥363 [19,32,33].

### 2.4. Scanning Electron Microscopy and Zeta Potential Analysis

**Figure 4:**
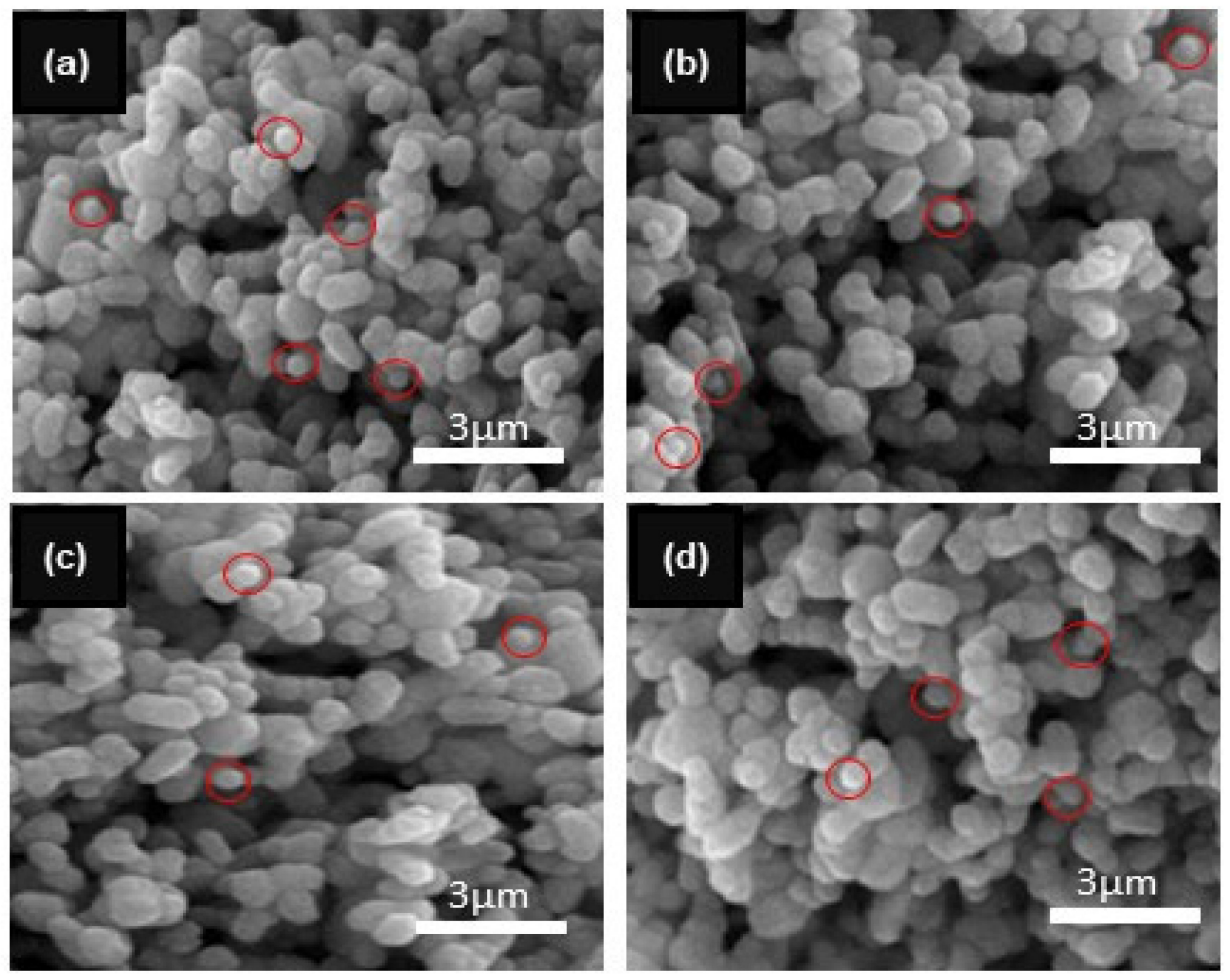
SEM results of (**a**) ZnO NPs and Al-doped ZnO NCs at varying concentrations (**b**) 0.25%, (**c**) 0.5%, and (**d**) 0.75%.

**Figure 5:**
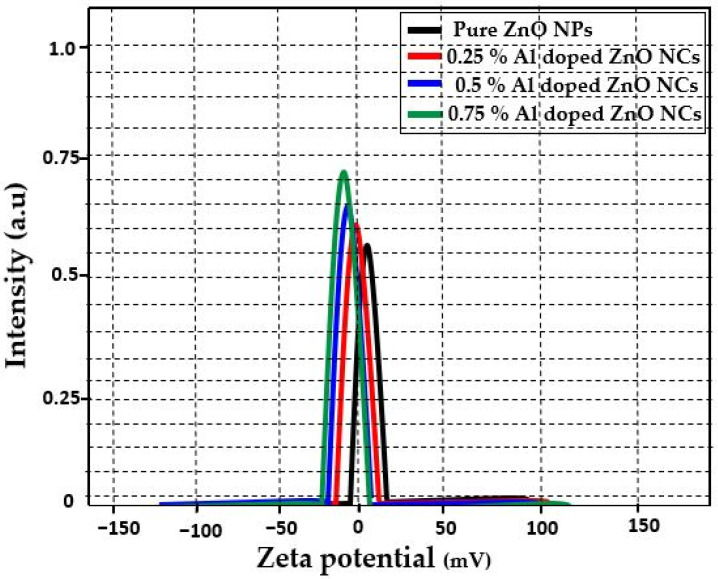
Zeta potential of ZnO NPs and Al-doped ZnO NCs at varying concentrations 0.25%, 0.5%, and 0.75%.

SEM analysis was conducted to investigate the surface morphology of the ZnO NPs and Al-doped ZnO NCs at varying concentrations (0.25%, 0.5%, and 0.75%), as shown in Figure 4a–d. With increasing Al^3+^ concentration, prominent changes in particle shape and size were observed. The SEM images demonstrate irregular shapes with small and large spherical grains, also observed in the Al-doped ZnO NCs. The spherical grains were circulated with red circles. In particular, the grain size increased with the increase in aluminum content. All the SEM images were taken at a resolution of 3 µm. Figure 4a–d displays SEM images of ZnO NPs, Zn_0.75_Al_0.25_O, Zn_0.5_Al_0.5_O, and Zn_0.25_Al_0.75_O NCs, revealing average crystallite sizes of 43.5, 40.5, 39, and 33 nm, respectively. Notably, these values are in excellent agreement with the corresponding XRD results, confirming the consistency of the crystallite size measurements. The zeta potential analysis results of ZnO NPs and Al-doped ZnO NCs are shown in Figure 5. The zeta potential values of ZnO NPs, Zn_0.75_Al_0.25_O, Zn_0.5_Al_0.5_O, and Zn_0.25_Al_0.75_O NCs dispersions were found to be +20, ±15, −10, and 13.7 mV, respectively. These results show the high stability of the prepared nanomaterials. These results are very similar to previously published articles [34,35,36].

### 2.5. Energy Dispersive X-ray Spectroscopy

**Figure 6:**
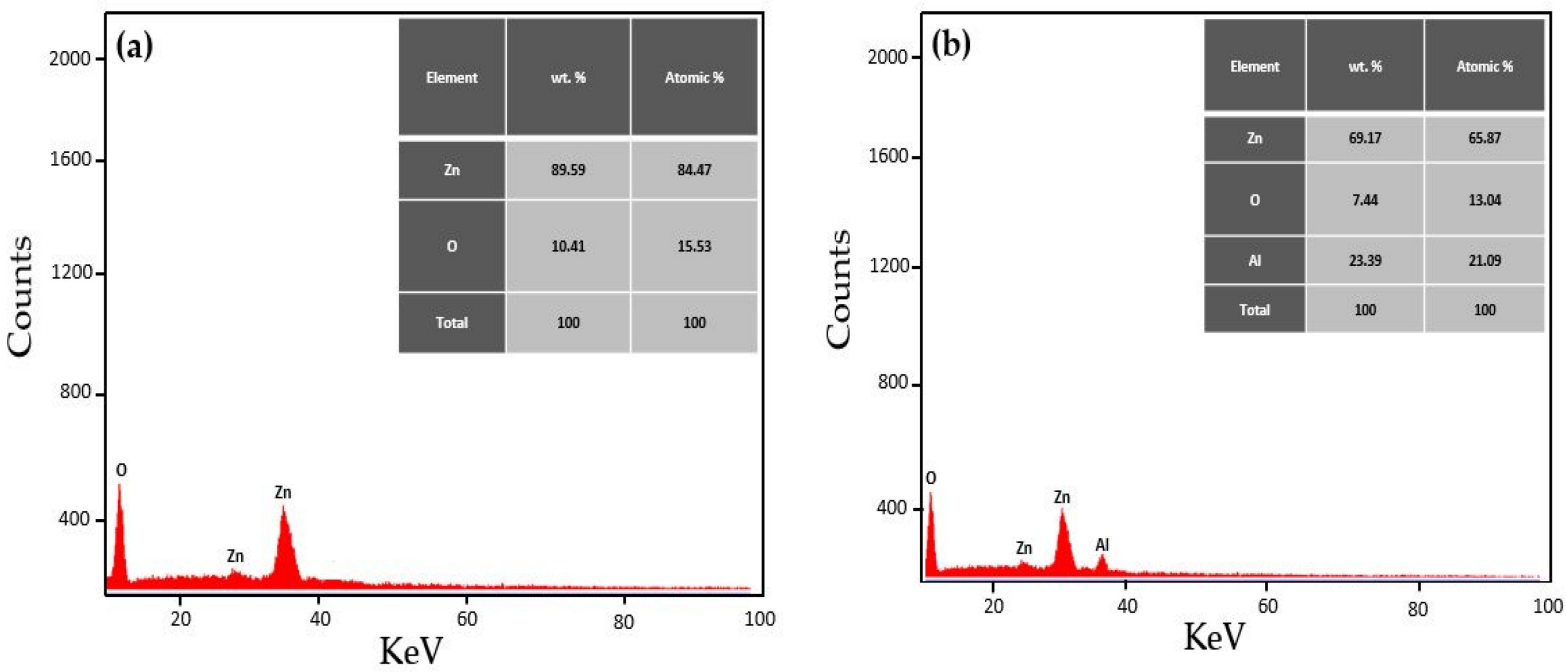

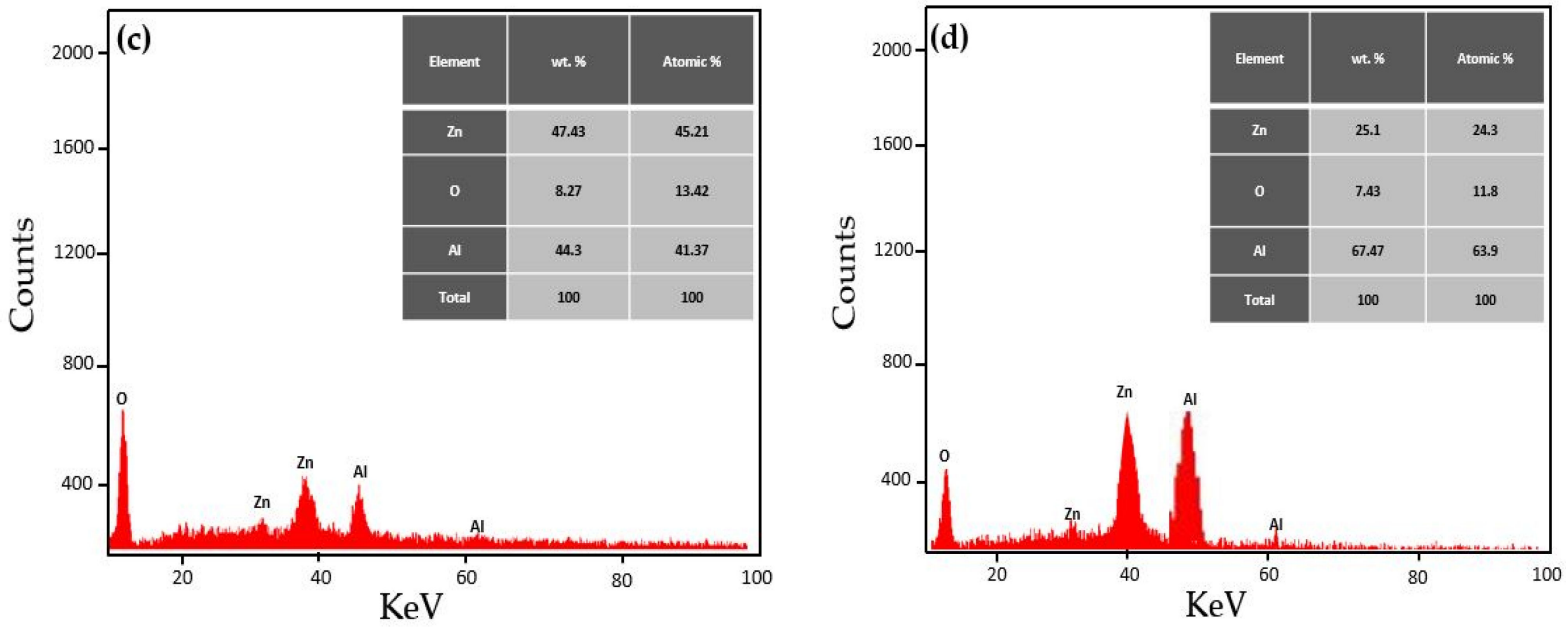
EDX spectrum of (**a**) ZnO NPs and Al-doped ZnO NCs at varying concentrations (**b**) 0.25%, (**c**) 0.5%, and (**d**) 0.75%.

The EDX analysis was employed to measure the elemental composition of the prepared ZnO NPs and Al-doped ZnO NCs. The outcomes of the EDX analysis confirmed the presence of Zn and Al as primary elements and O as a secondary element in the prepared nanomaterials, as shown in Figure 6a–d. For example, Musleh et al. [37] and Aldalbahi et al. [38] stated comparable outcomes where zinc and aluminum elements were detected in the EDX spectrum, confirming the reduction in zinc and aluminum-doped zinc ions to Zn and Al elements.

### 2.6. Antimicrobial Activity

**Figure 7:**
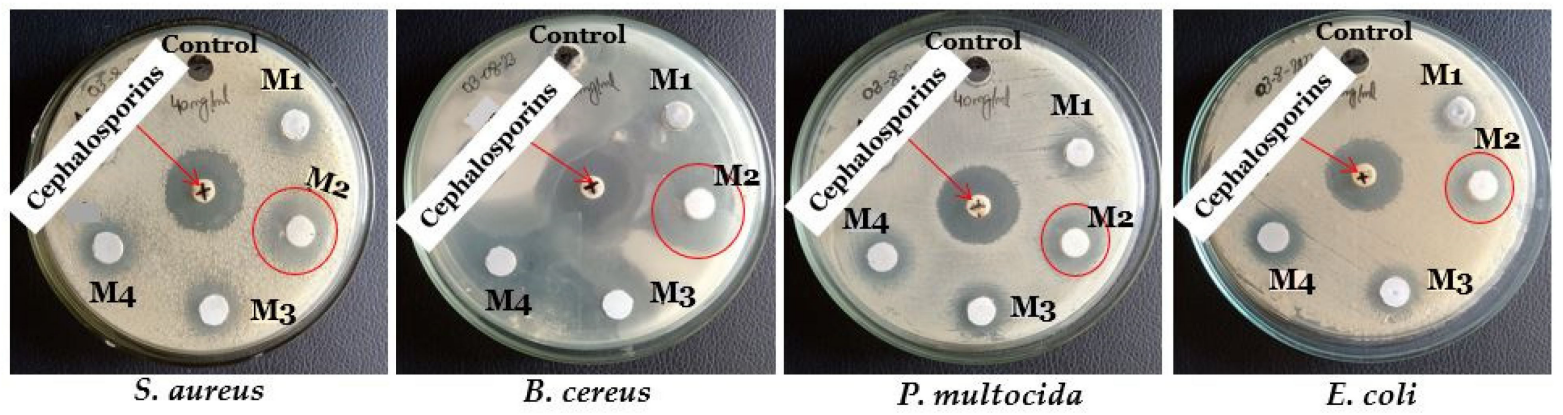
The antimicrobial activity of samples DMSO (control), standard antibiotic (cephalosporin), M1 (ZnO NPs), M2 (Zn_0.75_Al_0.25_O), M3 (Zn_0.5_Al_0.5_O), and M4 (Zn_0.25_Al_0.75_O NCs) against *S. aureus*, *B. cereus*, *P. multocida*, and *E. coli*.

**Figure 8:**
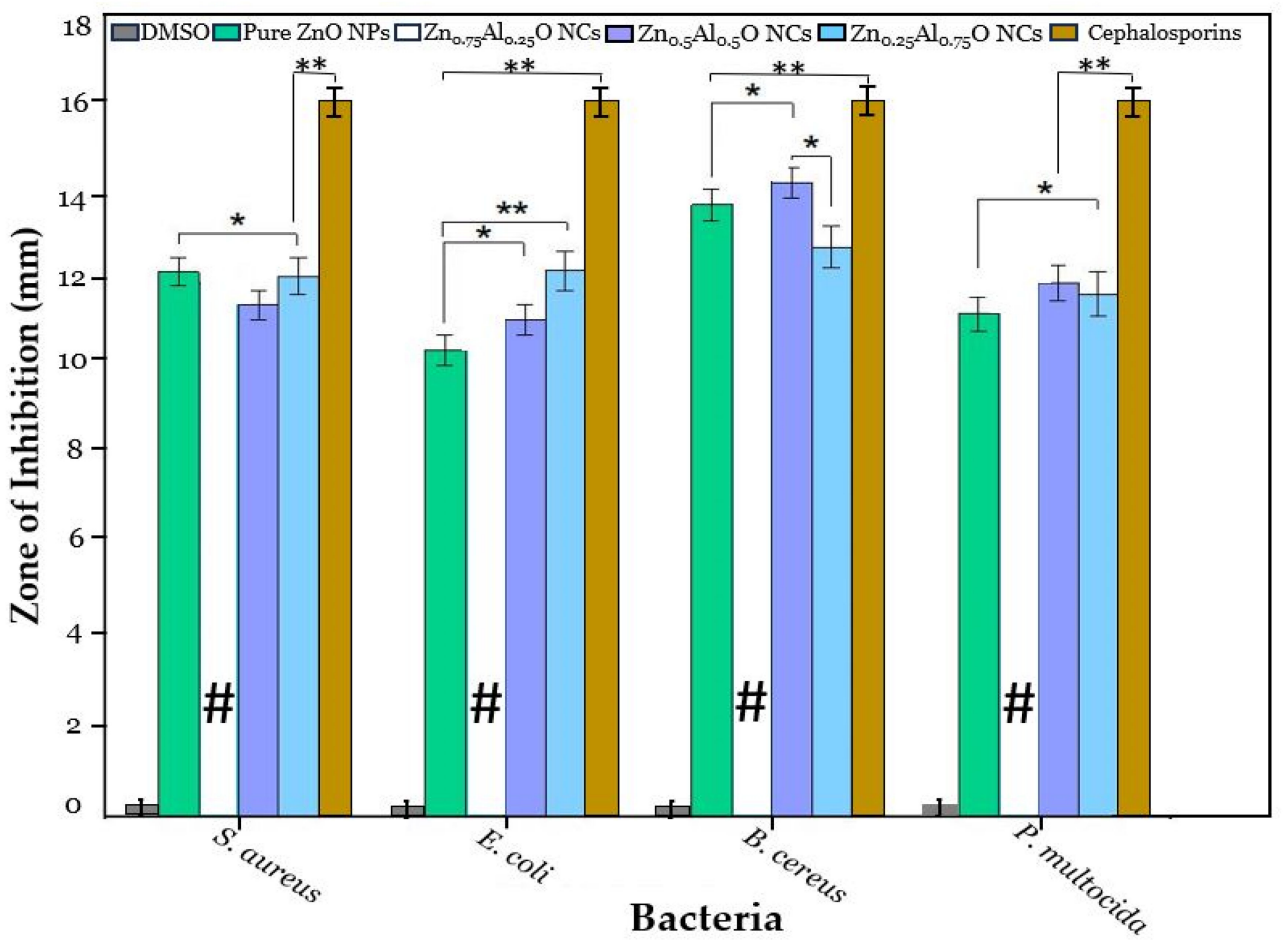
The antimicrobial activity of DMSO (control), standard antibiotic (cephalosporin), ZnO NPs, Zn_0.75_Al_0.25_O NCs, Zn_0.5_Al_0.5_O NCs, and Zn_0.25_Al_0.75_O NCs at 40 mg/mL, where * is significance for *p* ≤ 0.05 and ** is highly significance for *p* ≤ 0.01.

In this research work, the antimicrobial potential of ZnO NPs and Al-doped ZnO NCs at varying concentrations (0.25%, 0.5%, and 0.75%) was evaluated against four bacterial strains, including two Gram-positive strains, *Staphylococcus aureus* and *Bacillus cereus*, and two Gram-negative strains, *Escherichia coli*, and *Pasteurella multocida*. The results showed that ZnO NPs and Al-doped NCs exhibited the highest antimicrobial potential against the tested bacterial strains at a high-dose concentration of 40 mg/mL. Notably, the zones of inhibition were clear and significant, except for the Zn_0.75_Al_0.25_O NCs sample, which showed very high inhibition against all bacterial strains (indicated by the symbol “**#**”), as shown in Figure 7 and Figure 8.

**Figure 9:**
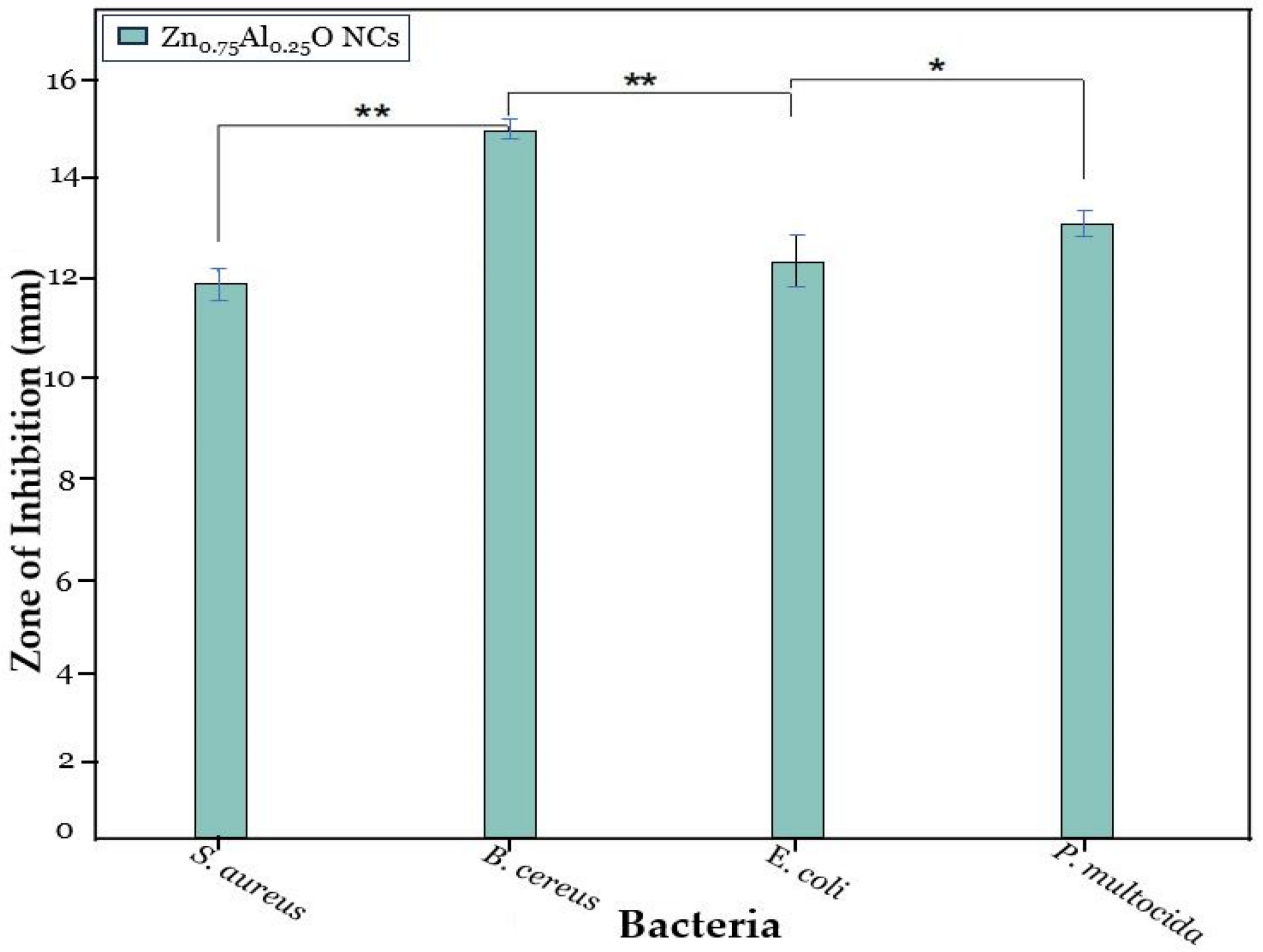
The antimicrobial activity of Zn_0.75_Al_0.25_O NCs at 150 mg/mL, where * is significance for *p* ≤ 0.05 and ** is highly significance for *p* ≤ 0.01.

Hence, the antimicrobial activity of Zn_0.75_Al_0.25_O NCs was repeated at lower concentrations, and even at 150 µg/mL, it showed a significant zone of inhibition against each bacterial strain, which showed its highest antimicrobial potential against these bacterial strains. A standard antibiotic, cephalosporin, was used as a positive control, and dimethyl sulfoxide (DMSO) was used as a negative control and did not show any antimicrobial activity against any of the bacterial strains to determine the assay validity. ZnO NPs, Zn_0.75_Al_0.25_O, Zn_0.5_Al_0.5_O, and Zn_0.25_Al_0.75_O NCs showed significant antimicrobial activity against all bacterial strains. Notably, Zn_0.75_Al_0.25_O NCs exhibited the highest antibacterial efficiency, especially against *B. cereus*. Moreover, Zn_0.75_Al_0.25_O NCs demonstrated the biggest inhibition zone against *S. aureus*, *B. cereus*, *E. coli*, and *P. multocida*, with 12 ± 0.09 mm, 15.5 ± 0.11 mm, 12.5 ± 0.07 mm, and 13.3 ± 0.11 mm, respectively, as shown in Figure 9 and Table 2. In this discussion, Yu et al. [23] described that Al-doped ZnO NCs showed great antibacterial activity against numerous bacterial strains, including *E. coli* and *P. aeruginosa*, especially against *S. aureus*. Also, it can decrease antimicrobial resistance (AMR). For instance, the antimicrobial activity of ZnO NPs can induce Zn^2+^ and ROS, such as hydroxyl radicals and superoxides, which can disrupt bacterial cell membrane integrity and DNA and inhibit enzyme activity, causing structural damage and leakage of cellular contents. These nanomaterials can prevent biofilm formation, which is important for bacterial survival and antibiotic resistance [38,39,40,41].

### 2.7. Anticancer Activity

**Figure 10:**
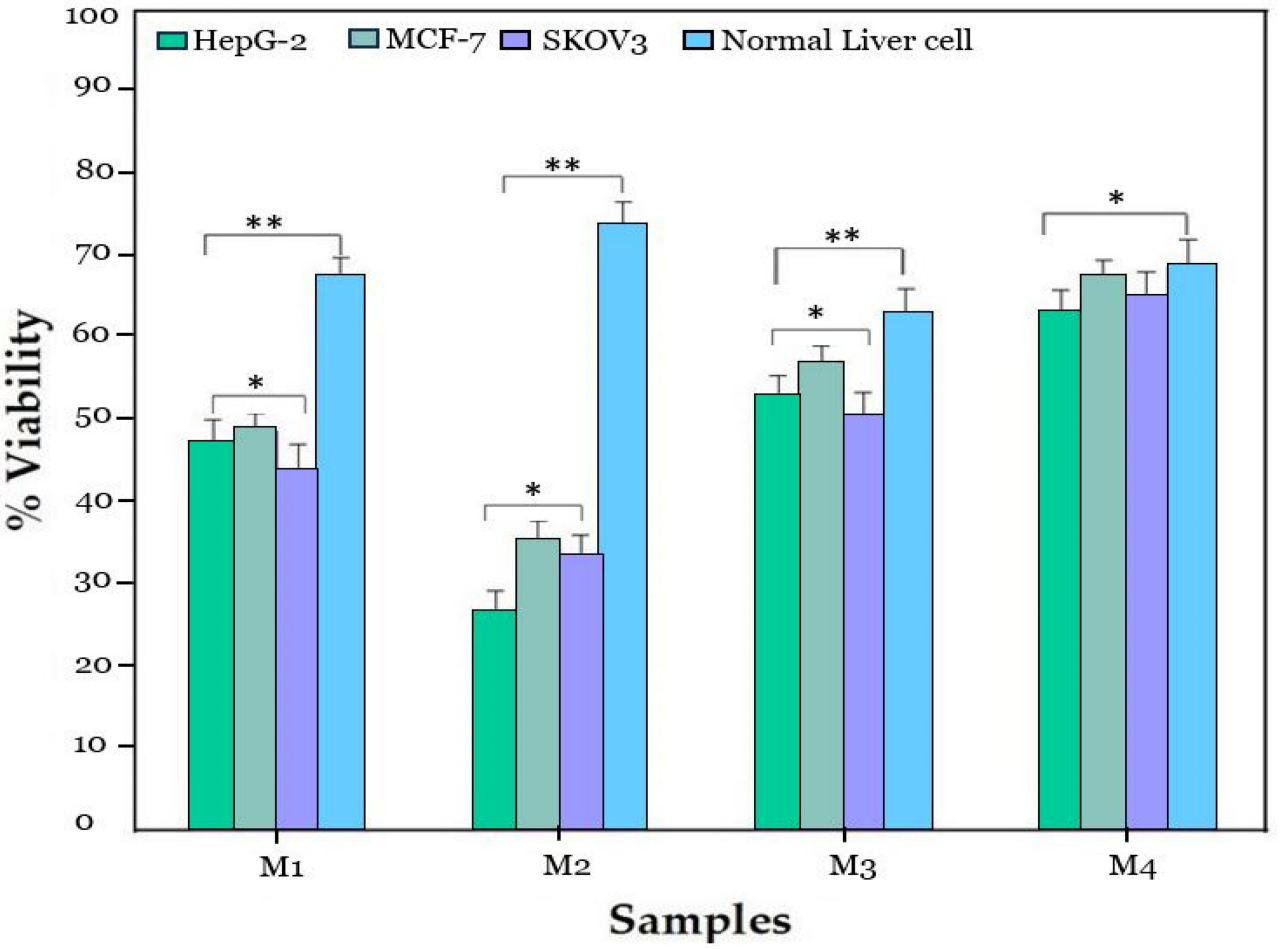
The anticancer activity of samples M1 (ZnO NPs), M2 (Zn_0.75_Al_0.25_O), M3 (Zn_0.5_Al_0.5_O), and M4 (Zn_0.25_Al_0.75_O NCs) against HepG-2, MCF-7, SKOV3 cancer cell lines, and normal liver cell line, where * is significance for *p* ≤ 0.05 and ** is highly significance for *p* ≤ 0.01.

**Figure 11:**
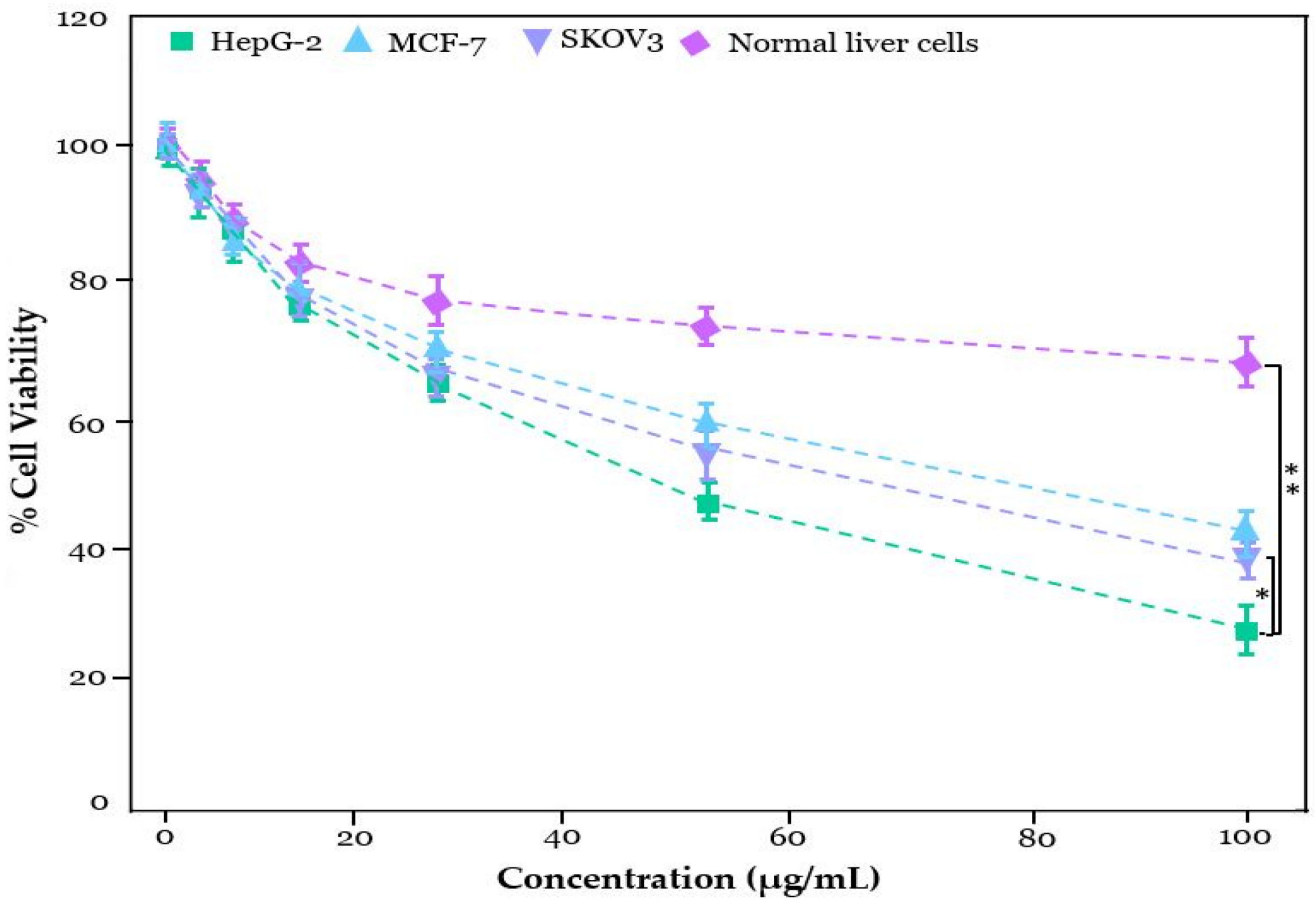
% cell viability of Zn_0.75_Al_0.25_O NCs at varying concentration (μg/mL) against HepG-2, MCF-7, SKOV3, and normal liver cell line, where * is significance for *p* ≤ 0.05 and ** is highly significance for *p* ≤ 0.01.

The 3-(4,5-dimethylthiazol-2-yl)-2,5-diphenyl-tetrazolium-bromide (MTT) assay was used to examine the cytotoxicity of ZnO NPs, Zn_0.75_Al_0.25_O, Zn_0.5_Al_0.5_O, and Zn_0.25_Al_0.75_O NCs toward HepG-2, MCF-7, SKOV3, and normal cells. In the initial screening, the cell viability of ZnO NPs, Zn_0.75_Al_0.25_O, Zn_0.5_Al_0.5_O, and Zn_0.25_Al_0.75_O NCs were tested against HepG-2, MCF-7, SKOV3, and normal cells at 100 μg/mL, and substantial cell viability was noticed for ZnO NPs and Zn_0.75_Al_0.25_O NCs compared to Zn_0.5_Al_0.5_O and Zn_0.25_Al_0.75_O NCs (see Figure 10). Notably, Zn_0.75_Al_0.25_O NCs exhibited > 70% cell viability toward HepG-2 cells. Zn_0.75_Al_0.25_O NCs showed reasonably higher % cell viability. For further validation, the antiproliferative activity of Zn_0.75_Al_0.25_O NCs was performed using a dose-dependent method (0, 3.125, 6.25, 12.5, 25, 50, and 100 µg/mL) toward all mentioned cell lines; then, dose-dependent curves were attained, as shown in Figure 11. The IC_50_ value was calculated as 20.47 µg/mL, indicating significant cell viability of Zn_0.75_Al_0.25_O NCs. The cell viability results showed that Zn_0.75_Al_0.25_O NCs not only enhanced HepG-2 cell destruction but also exhibited low cytotoxicity and high biocompatibility at low concentrations. Furthermore, Zn_0.75_Al_0.25_O NCs have reduced toxicity toward normal liver cells compared to HepG-2, MCF-7, and SKOV3 cells. These results suggest that Zn_0.75_Al_0.25_O NCs can serve as a promising preliminary point for further identification and isolation of compounds that inhibit HepG-2 cells or for the development of anticancer functional foods [34].

Allayeith et al. [41] described that ZnO NPs and their nanocomposites could be used for targeted cancer therapy, reducing side effects and improving the efficiency of cancer treatment. In these mechanisms, Al doping increases the surface area to volume ratio of ZnO and enhances the release of Zn^2+^ ions, improving interaction with cancer cells and bacterial strains (pathogens) and causing cell death and damage. Particularly, Al-doped ZnO NCs produce reactive oxygen species (ROS) and oxidative stress, leading to damage to cancer cell DNA, proteins, and lipids, as well as microbial damage. Anjum et al. [42] described that the anticancer activity of ZnO nanoparticles could induce programmed cell death (apoptosis) in cancer cells through mitochondrial dysfunction and activation of pro-apoptotic proteins. These nanoparticles can also inhibit cancer cell proliferation by arresting the cell cycle at specific stages (e.g., G2/M phase) and prevent the formation of new blood vessels, which is essential for cancer growth and metastasis. Notably, Al-doping can improve ROS generation, leading to enhanced antimicrobial and anticancer activity. Al-doping can facilitate the uptake of ZnO NPs by bacterial and cancer cells, enhancing their efficiency. Moreover, Al-doping can enhance the interaction between ZnO NPs and cell membranes, leading to superior structural damage.

## 3. Materials and Methods

### 3.1. Chemicals

Zinc nitrate [Zn(NO_3_)_2_, 98%], aluminum nitrate monohydrate [Al(NO_3_)_3_, 99.9%], sodium hydroxide (NaOH, 98%), MTT ≥ 98%, Dulbecco’s Modified Eagles Medium (DMEM), dimethyl sulfoxide (DMSO), fetal bovine serum (FBS), and nutrient agar were used as chemical reagents without any further purification. All chemicals were purchased from the Punjab Scientific store, Sigma Aldrich (St. Louis, MO, USA).

### 3.2. Synthesis of ZnO NPs and Al-Doped ZnO Nanocomposites

**Scheme 1:**
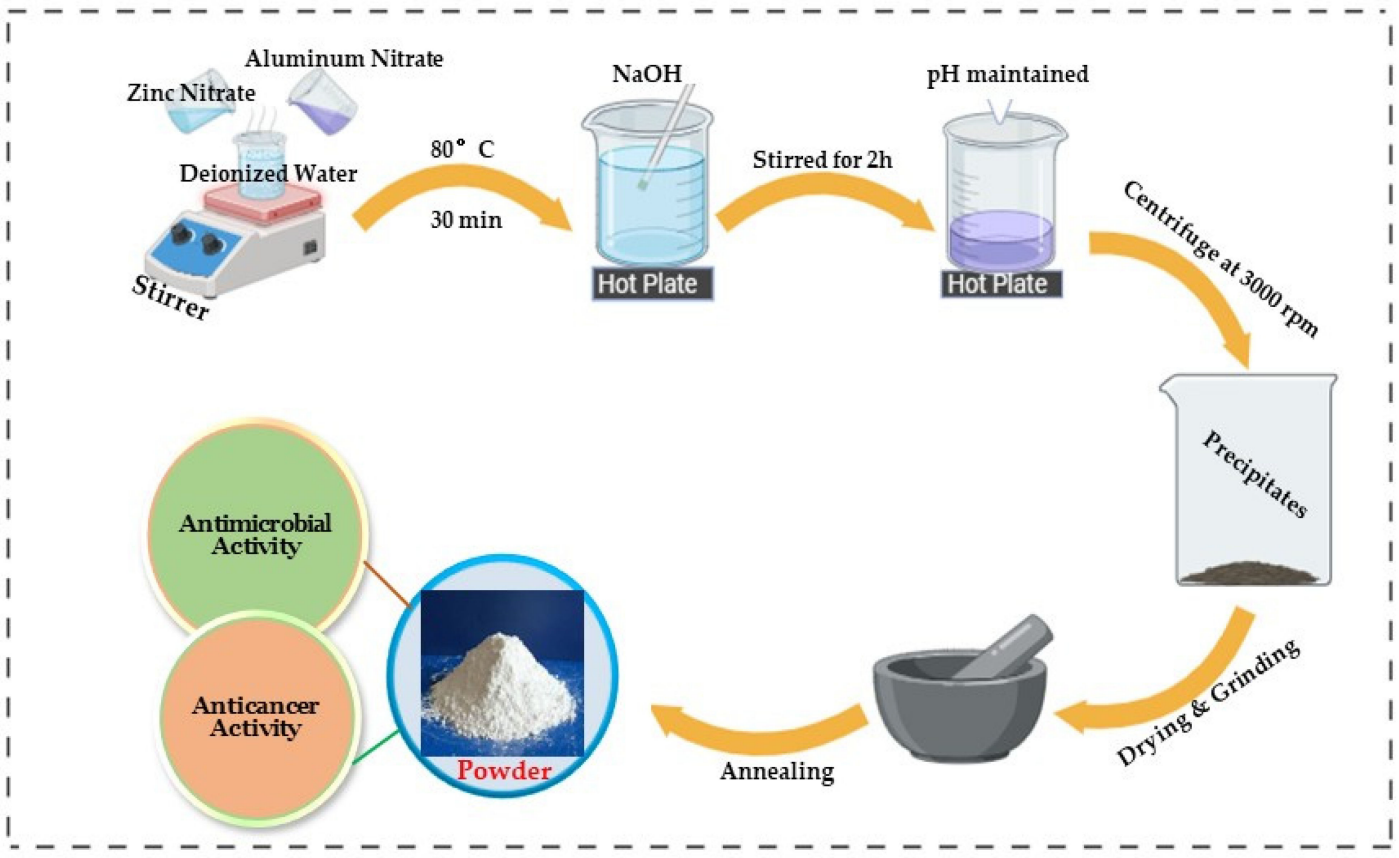
Synthesis process of ZnO NPs and Al-doped ZnO NCs.

A 0.5 M zinc nitrate solution was prepared using 100 mL of deionized water. In the next step, the solution was constantly stirred on a magnetic stirrer at 80 °C for 30 min. Furthermore, the pH was maintained between 10 and 11 by dropwise adding of NaOH solution. After 2 h of continuous stirring, a milky white precipitate formed. The precipitate was then centrifuged, washed, and dried. Finally, the ZnO NPs powder was annealed for 3 h at 300 °C in a muffle furnace. Similarly, Al-doped ZnO NCs were prepared by preparing a solution of 0.25% aluminum nitrate in 0.5 mM zinc nitrate. The solution was constantly stirred at 80 °C for 30 min. Finally, the same procedure was repeated twice with varying concentrations (0.5 and 0.75 wt%) [19,20]. The synthesis process of ZnO NPs and Al-doped ZnO NPs is shown in Scheme 1.

### 3.3. Characterization of Nanomaterials

SEM analysis was performed using the “Cube 10 Emcraft, Hanam, Republic of Korea” to analyze the surface morphology of the prepared nanomaterials. The UV-Vis spectrometer was employed to calculate the optical density on a “BK-UV1800PC double beam Bio-base, Jinan, China”. The XRD was performed using the “D8 advance Bruker, Karlsruhe, Germany” to examine the crystal structure using Cu Kα radiation (λ = 0.154). The FTIR analysis was performed on a “Spectrum 2, Perkin Elmer, Waltham, MA, USA” to analyze the functional groups. The EDX analysis was used to measure the elemental composition of the prepared nanomaterials on a “Zetasizer Nano ZS, Malvern, UK”.

### 3.4. Well Diffusion Assay

The *Staphylococcus aureus* (*S. aureus*), *Bacillus cereus* (*B. cereus*), *Pasteurella multocida* (*P. multocida*), and *Escherichia coli* (*E. coli*) strains were obtained from the laboratory of Muhammad Ali, Department of Zoology, Government College University Faisalabad, Pakistan. The antimicrobial potential of ZnO NPs and Al-doped ZnO NCs was evaluated using a modified well diffusion method [21]. Nutrient agar powder (2.8 g) was dissolved in distilled water (100 mL). The media was then poured into sterile petri dishes in a biosafety cabinet and allowed to solidify for 2 hours. The plates were stacked and sealed, then incubated overnight at 37 °C to check for pre-experimental contamination. After 24 h, 100 µL of each strain (*E. coli*, *B. cereus*, *S. aureus*, and *P. multocida*) was inoculated onto agar plates at a concentration of 0.6 McFarland scale. Solutions of ZnO NPs and Al-doped ZnO NPs (40 mg/mL) were dispensed into separate plates in the agar medium and incubated at 37 °C for 24 h. Cephalosporin antibiotics were used as a positive control, while DMSO was used as a negative control to determine the validity of the assay. After 24 h, the inhibition zones were measured using a vernier caliper.

### 3.5. Cell Culturing

Liver cancer (HepG-2), breast cancer (MCF-7), ovarian cancer (SKOV3), and normal liver cell lines were gifted from the laboratory of Muhammad Ali, Department of Zoology, Government College University Faisalabad (GCUF), Pakistan. HepG-2, MCF-7, SKOV3, and normal liver cells were cultured in a 96-well plate separately, using a culture medium supplemented with FBS (10%), DMEM (2 mM), penicillin and streptomycin (100 IU mL^−1^), as well as HEPES (20 mM) as a buffering medium. The mixture was maintained under optimal conditions (37 °C, 5% CO_2_) in a humidified atmosphere. The standard protocol was adopted as recommended by the Ethics Committee of GCUF. For more details, see our latest published paper [5,20].

### 3.6. MTT Assay

Using the MTT assay, the in vitro cytotoxicity of ZnO NPs and Al-doped ZnO NCs on the HepG-2, MCF-7, SKOV3, and normal liver cell lines was evaluated. HepG-2, MCF-7, SKOV3, and normal liver cells were separately cultured in a 96-well plate and incubated at 37 °C for 24 h. Then, the cells were incubated for 24 h in varying concentrations of nanocomposites for treatment. After 24 h, 10 microliters (µL) of MTT reagent was added, and the mixture was incubated for an additional 4 h. Subsequently, 150 microliters (µL) of DMSO was used to dissolve the formazan crystals, and the absorbance was determined using a microplate reader [28].

Equation (2) was used to calculate cell viability.

(2) $$\%\;Cell\;Viability=\frac{{Absorbance}_{C}-{Absorbance}_{T}}{{Absorbance}_{C}}\times 100\;\%$$

### 3.7. Statistical Analysis

The statistical analysis was performed using GraphPad Prism (version 8.0). The mean ± standard deviation of each experiment is shown, with * *p* < 0.05 indicating statistical significance, and ** *p* < 0.01 indicating high statistical significance.

## 4. Conclusions

Zinc oxide nanoparticles and aluminum-doped zinc oxide nanocomposites were prepared using the coprecipitation method. The XRD outcomes revealed a hexagonal wurtzite phase structure. UV-Vis results determined maximum absorption spectra at 363 nm and ≥363, respectively, confirming the complete formation of ZnO NPs and Al-doped ZnO NCs. Zeta potential values of ZnO NPs, Zn_0.75_Al_0.25_O, Zn_0.5_Al_0.5_O, and Zn_0.25_Al_0.75_O NCs were found to be +20, ±15, −10, and 13.7 mV, respectively. The SEM micrograph results revealed nonuniform, agglomerated, irregularly shaped particles with small and large spherical grains. With increasing Al^3+^ concentration, prominent changes in particle shape and size were observed. Additionally, an in vitro MTT assay of ZnO NPs and Al-doped ZnO NCs disclosed that they possessed significant anticancer activity toward HepG-2, MCF-7, SKOV3, and normal liver cells, respectively. The microbial activity of ZnO NPs and Al-doped ZnO NCs was also examined by well diffusion assay toward *S. aureus*, *B. cereus*, *E. coli*, and *P. multocida*. However, the Zn_0.75_Al_0.25_O NCs exhibited the highest inhibition zone against *B. cereus*. Additionally, Zn_0.75_Al_0.25_O NCs not only enhanced the destruction of cancerous cells but also showed low toxicity and high biocompatibility at low concentrations. These findings indicate that Zn_0.75_Al_0.25_O NCs could serve as a new inhibitor for cytotoxic and antimicrobial agent, which suggests that it could be a promising candidate for in vivo anticancer and antimicrobial applications.

## Figures and Tables

**Table 2:** Inhibition of ZnO NPs and Al-doped ZnO NCs at varying concentrations (0.25%, 0.5%, and 0.75%) against *S. aureus*, *E. coli*, *B. cereus*, and *P. multocida*.

Samples	*S. aureus*	*E. coli*	*B. cereus*	*P. multocida*
DMSO	0 ± 0.03 mm	0 ± 0.03 mm	0 ± 0.03 mm	0 ± 0.03 mm
M1	12 ± 0.05 mm	10.2 ± 0.07 mm	13.9 ± 0.08 mm	11.1 ± 0.08 mm
M2	12 ± 0.09 mm	12.5 ± 0.07 mm	15.5 ± 0.11 mm	13.3 ± 0.11 mm
M3	11.5 ± 0.07 mm	10.9 ± 0.09 mm	14.2 ± 0.08 mm	11.9 ± 0.09 mm
M4	12 ± 0.07 mm	12.2 ± 0.09 mm	13 ± 0.09 mm	11.8 ± 0.11 mm
Cephalosporins	16 ± 0.07 mm	16 ± 0.07 mm	16 ± 0.07 mm	16 ± 0.07 mm

## Data Availability

Data is contained within the article.
